# Screen-Printed Carbon Electrode Modified with Carbon Nanotubes and Copper Film as a Simple Tool for Determination of Trace Concentrations of Lead Ions

**DOI:** 10.3390/membranes14020053

**Published:** 2024-02-12

**Authors:** Malgorzata Grabarczyk, Agnieszka Wawruch

**Affiliations:** Department of Analytical Chemistry, Institute of Chemical Sciences, Faculty of Chemistry, Maria Curie-Sklodowska University, 20-031 Lublin, Poland; wawruch.agnieszka@gmail.com

**Keywords:** lead, copper film electrode, carbon nanotube-modified screen-printed carbon electrode, stripping voltammetry

## Abstract

A copper film-modified, carboxyl-functionalized, and multi-walled carbon nanotube (MWCNT-COOH)-modified screen-printed carbon electrode (CuF/MWCNTs/SPCE) was used for lead determination using anodic stripping voltammetry. The main parameters were investigated and optimized during the development of the research procedure. The most optimal electrolyte concentrations were determined to be 0.4 M HCl and 6.3 × 10^−5^ M Cu(II). The optimal parameters for voltammetric stripping measurements are as follows: an accumulation potential of −0.7 V; an accumulation time of 120 s; and a pulse amplitude and pulse time of 120 mV and 2 ms, respectively. The effect of surface active substances and humic substances as potential interferents present in aqueous environmental samples was investigated. The validation of the procedure was carried out using certified reference materials, like waste water SPS-WW1 and environmental matrix TM-25.5. In addition, the developed procedure was applied to investigate lead recovery from natural environmental water, such as rivers and lakes.

## 1. Introduction

Lead plays a key role in various industrial processes, but its accumulation above acceptable levels poses a serious threat to human health. The release of lead into the environment, from electroplating to metallurgical processes or the paper industry, poses a significant risk of contamination, especially to crops through its accumulation in water and soil. Lead, along with cadmium, stands out as particularly toxic, leading to a range of health problems, such as cancer, nervous system disorders, liver dysfunction, and cardiovascular problems. The tolerable weekly intake of lead with food in humans is 25 mg/kg body weight (WHO, 2010). According to Polish law (Regulation of the Minister of Health, 2017), the maximum concentration of lead in water intended for consumption is 10 μg/L [1,2]. For this reason, analytical methods to determine the amount of lead in the environment are very important. There are known procedures for determining lead by flame atomic absorption spectrometry (FAAS) or inductively coupled plasma mass spectrometry (ICP-MS), but these are not methods that can be easily or cheaply used under field conditions [3,4,5,6,7]. Electrochemical methods, such as stripping voltammetry, are far better for such applications [8].

Electrochemical methods offer the benefits of elevated sensitivity, accuracy, and low cost; further, they are simple to use, and their instruments are relatively portable [9]. There are many papers devoted to the determination of lead by voltammetric sensors [10,11,12]. The most popular method is anodic stripping voltammetry, which is applied to different samples using different working electrodes. Formerly, mercury electrodes were mainly used, while currently, the most common ones are carbon-based electrodes, which are modified with metal films or by adding modifiers to carbon paste [8,13,14]. Film electrodes are distinguished by their favorable surface-to-volume ratio, which leads to increased sensitivity. The most common film-forming metals are bismuth, lead, antimony [1,15,16,17], and now also copper [18,19,20]. The surface of the electrode can also be modified with carbon nanomaterials, and the range of materials being modified is constantly expanding. Nanomaterials improve electrode properties that are important in voltammetric measurements; for example, they increase the surface area of the working electrode, exhibit excellent electrical conductivity, and have good chemical stability [21]. There has already been a lot of work on the determination of metal ions on an electrode modified with carbon nanotubes [22,23,24,25,26,27]. Another important aspect in the selection of a working electrode, apart from its composition, is its design.

Screen-printed electrodes have become more and more popular in recent years. They are easily and cheaply produced in large quantities and much more convenient to use under environmental conditions, since one small strip contains the three electrodes necessary for voltammetric measurements [19,28,29]. They are a very good solution for field measurements, as they enable the measurement of a water sample immediately after it is collected in the field. Their surface can be modified with metal films or nanoparticles as in conventional electrodes. The range of screen-printed electrodes produced is very wide. For example, there are screen-printed electrodes in which the role of the working electrode is usually played by carbon, gold, platinum, silver, or carbon nanotubes, but there are also SPEs with palladium or ruthenium oxide. Also available are electrodes with the surface modified by the manufacturer with a thick film of metal, such as antimony, chromium, aluminum, lead, or cobalt, and there are also boron-doped diamond working electrodes [30]. In our work, in order to increase the sensitivity of Pb(II) determinations and develop a procedure that allows for quick and simple analysis of environmental samples, a carboxyl-functionalized, multi-walled carbon nanotube (MWCNT-COOH)-modified, screen-printed electrode, which was further modified by applying a copper film, was used for the study. Such an electrode has previously been successfully used by us for the determination of trace amounts of Cd(II) ions in aqueous environmental samples [19]. The novelty of the work is the use of an electrode described for the first time in [19] for the determination of trace amounts of Pb(II). The developed copper film-modified, carboxyl-functionalized, and multi-walled carbon nanotube-modified screen-printed electrode (CuF/MWCNTs/SPCE) provided a very low detection limit for Pb(II), which is confirmed by the data collected in Table 1, showing the most important parameters of the procedures developed in recent years for the determination of Pb(II) using, for example, a modified glassy carbon electrode (GCE), a modified screen-printed electrode (SPE), a modified carbon paste electrode (CPE), or a boron-doped nanodiamond electrode (BDND) as the working electrode. As can be seen from Table 1, both screen-printed and carbon nanotube electrodes have previously been used to determine trace amounts of Pb(II). However, in our work, an MWCNT/SPCE coated with a copper film was used for the first time, resulting in the lowest detection limits shown in Table 1. In addition, a great advantage of the proposed procedure is its insensitivity to surfactants and humic substances, the effects of which have been precisely studied.

## 2. Materials and Methods

### 2.1. Apparatus

To perform voltammetric studies, a µAutolab analyzer (EcoChemie, Utrecht, The Netherlands) was used. DropSens DRP-110CNT electrodes (Metrohm AG, Herisau, Switzerland) were used for measurements. According to the manufacturer’s technical specifications, these electrodes were screen-printed carbon electrodes (SPCEs) modified with carboxyl-functionalized multi-walled carbon nanotubes (MWCNT-COOH). The working electrode was MWCNT-COOH, the auxiliary electrode was carbon, and the reference electrode was silver. Before each measurement, the surfaces of the working electrodes were modified in situ with copper. The voltammetric cell used had a volume of 10 mL. To take images of the electrode surfaces, a Quanta 3D FEG (FEI Company, Hillsboro, Oregon, USA) scanning electron microscope (SEM) with an EDS attachment was used.

### 2.2. Reagents

Standard solutions of Pb(II) and Cu(II) at 1 g/L were obtained from Fluka (Buchs, Switzerland). Working solutions of 1 × 10^−4^ M, 1 × 10^−6^ M, and 1 × 10^−7^ M for Pb(II), and 1 × 10^−4^ M for Cu(II) were prepared from the standard solutions. They were acidified with 0.005 M HCl. Concentrated hydrochloric acid (Suprapure, Merck, Darmstadt, Germany) diluted to 0.4 M was used as a supporting electrolyte. The effect of surfactants was studied using the non-ionic surfactant Triton X-100, the anionic surfactant SDS, and the cationic surfactant CTAB, all purchased from Fluka. The effects of humic acids (HAs, Aldrich, Darmstadt, Germany), fulvic acids (FAs), and natural organic matter (NOM) (both from the International Humic Substances Society, Denver, Colorado, USA) were also studied. The certified reference materials used were waste water SPS-WW1 (Spectrapure Standards As, Oslo, Norway) and the environmental matrix reference material TM-25.5 (Environment and Climate Change, Ottawa, ON, Canada).

### 2.3. ASV Procedure of Lead Determination

**Figure 1:**
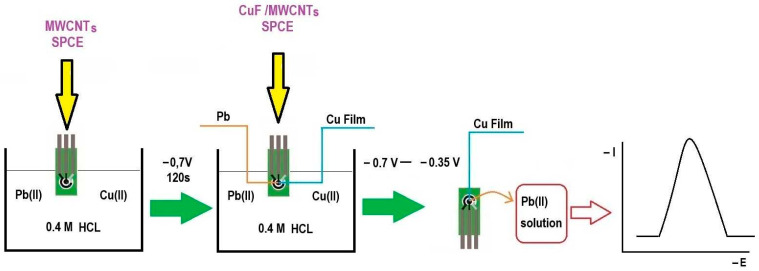
A scheme illustrating the sensing principle of the proposed electrode.

Measurements were performed under optimized conditions. In the measurement solution, the concentration of the supporting electrolyte was 0.4 M and the concentration of Cu(II) was 6.3 × 10^−5^ M. For voltammetric measurements, the following parameters were chosen: Before each measurement, the electrode was cleaned with the electrochemical method by applying a potential of +0.4 V to it for 10 s and then setting the accumulation potential to −0.7 V, with an accumulation time of 120 s. At this stage, copper and lead were simultaneously deposited on the electrode surface. After this step, the signal obtained when the potential of the screen-printed electrode was changed from −0.7 V to −0.35 V was recorded. The sensing principle of the proposed electrode is illustrated in Figure 1. On the recorded voltammograms, the peak height was directly proportional to the concentration of Pb(II) ions in the solution. The potential range used during signal recording was within the permissible potential window for the electrode used. The permissible potential range is −0.8 V on the cathode side, being limited by the reduction of hydrogen ions, and −0.4 V on the anode side, being limited by copper oxidation (see Figure 7 in [19]).

### 2.4. Preparation of Certified Reference Materials and Real Samples

The SPS-WW1 and TM-25.5 reference materials used were significantly acidified by their manufacturers. When testing the above materials, 0.2 mL of SPS-WW1 and 1 mL of TM-25.5 were taken into the voltammetric cell. In order to neutralize the acid present in these materials, 20 μL and 31.5 μL 2 M NaOH, respectively, were also added to the test solutions. This was necessary in order not to have too low a pH, at which the signal would have been obtained at a different potential.

Tests were also conducted on real water samples. Water samples from the Bystrzyca River and Lake Piaseczno (located in Lublin and in Kaniwola, respectively, in east Poland) were taken into a glass container. They were stored at 5 °C. When conducting tests on natural water samples, it is important to keep in mind that they may contain surfactants and organic substances that can adhere to the surface of the electrode, blocking it and reducing the sensitivity of the determinations. To prevent these processes, Amberlite XAD-7 resin can be used. In the case of our method, 0.1 g of resin was added to the measuring vessel with 10 mL of river water; then, it was stirred, and after 10 min a 1 mL sample was taken for further testing.

## 3. Results and Discussion

Optimization of the conditions for the determination of lead on a screen-printed electrode with carbon nanotubes and a copper film generated on it was carried out. The following parameters were optimized: the concentration of the supporting electrolyte, the concentration of Cu(II), the potential and accumulation time, and the pulse height and pulse time in the differential pulse stripping voltammetry method.

### 3.1. Morphology and Composition of the Electrode Material

**Figure 2:**
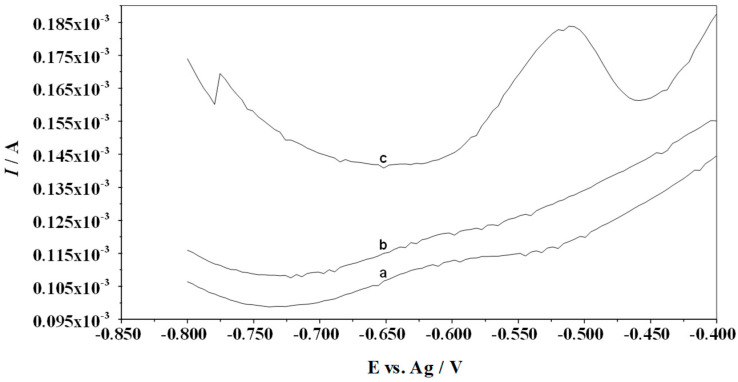
Voltammograms recorded for the solutions containing the following: (a) background: 4 mL 1 M HCl; (b) as (a) +1 × 10^−7^ M Pb(II); (c) as (b) +7 × 10^−5^ M Cu(II). Accumulation potential −0.8 V and accumulation time 60 s. Scan rate 40 mV/s. Pulse time 2 ms and pulse height 120 mV.

As experimentally demonstrated, there is no signal from lead in the case of the test solution containing no Cu(II) because lead does not concentrate directly on the surface of the MWCNTs/SPCE electrode. Figure 2 shows the voltammograms for the solution with lead and without Cu(II), and after Cu(II) was added to the solution. The background is the voltammetry recorded in the base electrolyte solution in the absence of a depolarizer, which in our work is Pb(II). The peak from the lead ions appears only when Cu(II) ions are present in the solution. This is indicated by the formation of a copper (Cu) film on the surface of the electrode, which allows the accumulation of lead.

**Figure 3:**
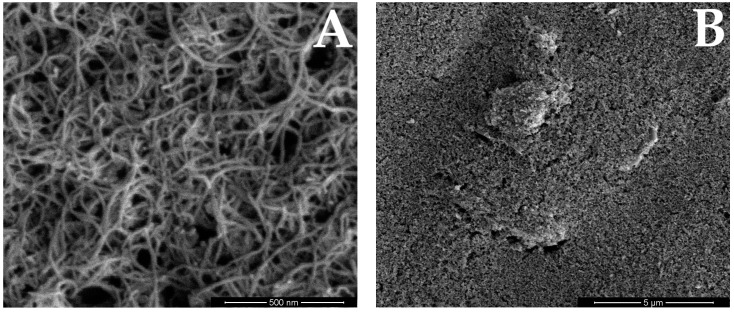

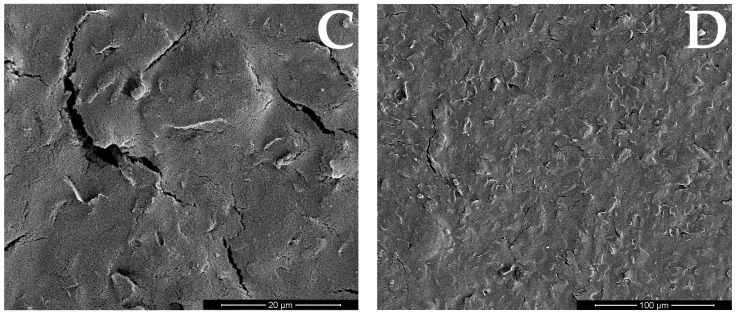
The SEM images of the surface of the working electrode. The photos were taken at the following magnifications: (**A**) 100,000×, (**B**) 10,000×, (**C**) 2500×, (**D**) 500×.

**Figure 4:**
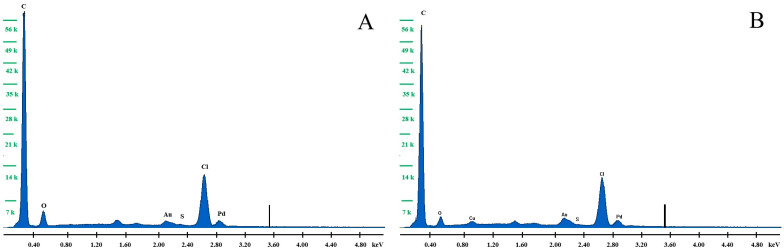
The EDS spectra obtained for the unmodified CuF/MWCNTs/SPCE electrode (**A**) and after modification (**B**) with a copper film.

SEM images showing the surface morphology of the working electrode were taken and they are shown in Figure 3. The carbon nanotubes with which the screen-printed electrode was modified can be seen in detail. No clusters of copper, whose film was applied to the electrode, can be seen, but the SEM was coupled with an EDS, so the presence of copper was confirmed by the EDS analysis performed. Table 2 shows the results of this analysis obtained for the unmodified CuF/MWCNTs/SPCE electrode and after modification with a copper film. Figure 4 shows the EDS spectra. It can be noted that the unmodified electrode does not contain copper, whereas the electrode after modification definitely contains copper in its composition.

### 3.2. Influence of Supporting Electrolyte Concentration

Based on previous work on the determination of metal ions on the CuF/MWCNTs/SPCE, hydrochloric acid was chosen as the supporting electrolyte [19]. Tests were conducted on a solution containing 1.2 × 10^−5^ M Cu (II), 5 × 10^−8^ M Pb(II), and an appropriate concentration of HCl. The concentrations tested ranged from 0.05 M to 0.4 M. It was observed that over the entire concentration range tested, the peaks obtained were of comparable height and shape. Bearing in mind that the method is to be used for testing real samples, the highest concentration was chosen as the most optimal one, and at the same time providing the most stable pH during the testing of real samples. All subsequent measurements were carried out at a HCl concentration of 0.4 M in the test solution.

### 3.3. Influence of Cu(II) Concentration

**Figure 5:**
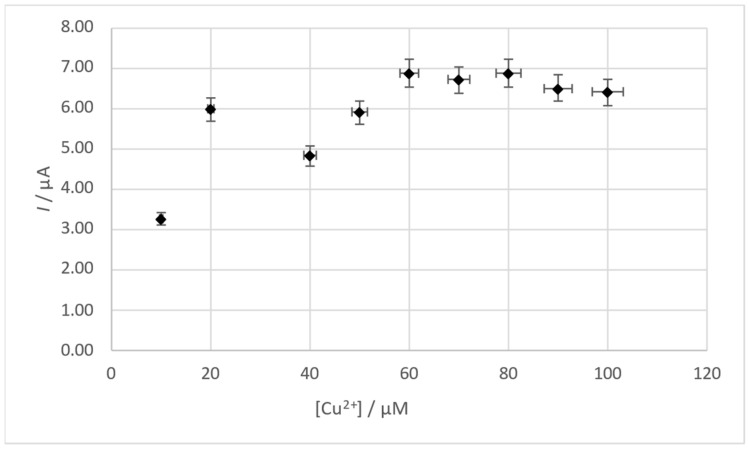
Influence of Cu(II) concentration on the Pb(II) signal. Concentration of Pb(II) 1 × 10^−8^ M. Accumulation potential −0.8 V and accumulation time 60 s. Scan rate 40 mV/s. Pulse time 2 ms and pulse height 50 mV.

The next parameter whose influence was studied was the concentration of the film-forming metal — copper. A solution containing 0.4 M HCl and 1 × 10^−8^ M Pb(II) was prepared and then successive portions of Cu(II) were added with an initial concentration of 1 × 10^−5^ M. After each Cu(II) addition, the voltammogram was recorded and the lead peak current was measured. A range of Cu(II) concentrations from 1 × 10^−5^ M to 1 × 10^−4^ M was tested. It was proven that Cu(II) concentration affects the signal from lead. Without the addition of Cu(II), no signal from lead is observed, and then there is a noticeable increase in the signal in the Cu(II) concentration range from 1 × 10^−5^ M to 6 × 10^−5^ M. At a Cu(II) concentration between 6 × 10^−5^ M and 8 × 10^−5^ M, the signal is practically constant, whereas at higher concentrations it begins to slowly decrease. The influence of Cu(II) concentration on the Pb(II) peak current is presented in Figure 5. An addition of Cu(II) at 1g/L to a 40 μL standard solution was used for subsequent tests, which resulted in a concentration of 6.3 × 10^−5^ M Cu(II) in the test solutions.

### 3.4. Conditions of Accumulation Potential and Time

**Figure 6:**
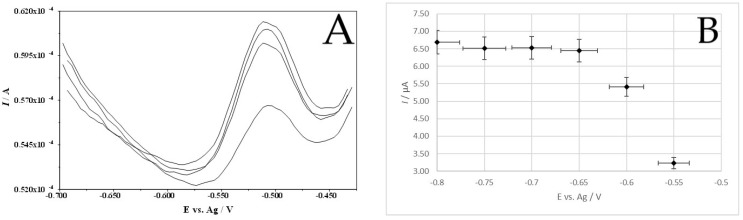
Example voltammograms (**A**) and the influence of accumulation potential (**B**) on the Pb(II) signal. Composition of the solution: 1 × 10^−7^ M Pb(II), 6.3 × 10^−5^ M Cu(II), and 0.4 M HCl. Scan rate 40 mV/s. Pulse time 2 ms and pulse height 50 mV.

When a correspondingly negative accumulation potential is applied to the CuF/MWCNTs/SPCE, a copper film is formed on the electrode and Pb(II), reduced to Pb(0), is simultaneously deposited. Therefore, a series of measurements were carried out to select an appropriate potential value in order to obtain the highest lead peak. The measurements were carried out on a solution containing 1 × 10^−8^ M Pb(II), 6.3 × 10^−5^ M Cu(II), and 0.4 M HCl. Accumulation potentials in the range from −1.1 V to −0.5 V were checked for an accumulation time of 60 s. The voltammograms obtained allow us to conclude that the best potential for obtaining high and symmetrical peaks is −0.7 V. At more negative potentials, the peaks are lower; at between −0.8 V and −0.65 V, they are comparable, and then they decrease sharply again. Example voltammograms are presented in Figure 6A, while the influence of accumulation potential is presented in Figure 6B.

**Figure 7:**
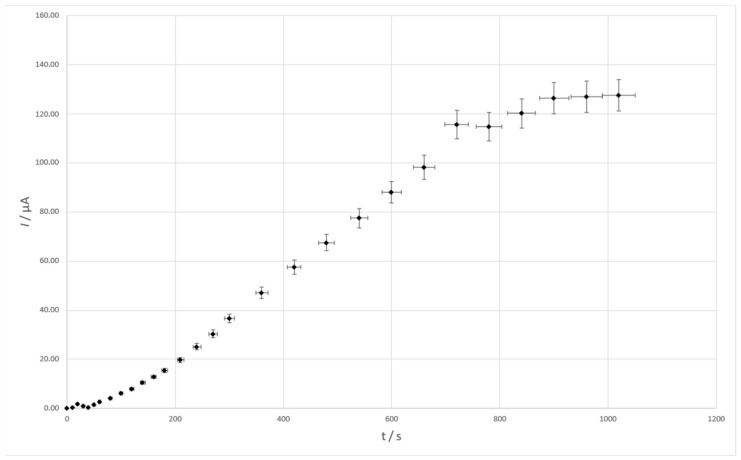
The influence of accumulation time on the Pb(II) signal. Composition of the solution: 1 × 10^−7^ M Pb(II), 6.3 × 10^−5^ M Cu(II), and 0.4 M HCl. Scan rate 40 mV/s. Pulse time 2 ms and pulse height 120 mV.

The timing of this stage is equally important for accumulation. The magnitude of the lead signal that can be obtained was tested by conducting tests on a solution containing 2 × 10^−8^ M Pb(II), 6.3 × 10^−5^ M Cu(II), and 0.4 M HCl at accumulation times ranging from 0 to 900 s. The results show that the longer the time, the higher the peak current, but also the slope of the peak current vs. time curve decreases for accumulation times above 600 s. The influence of accumulation time on the Pb(II) peak current is presented in Figure 7.

Electrochemical cleaning parameters were also investigated. To this end, a suitably positive potential was applied to the working electrode for a time of 10 s before each measurement in order to remove any residue from the previous measurement. The tests were carried out using potentials ranging from 0 to +0.4 V. It was found that in each case the lead signal recorded after such cleaning was comparable, indicating that the electrode surface had been effectively cleaned. A potential of +0.4 V and a time of 10 s were used as standard cleaning parameters between measurements in subsequent experiments.

### 3.5. Optimization of DPASV Parameters

The DP technique parameters such as pulse time and pulse height were also optimized. Measurements were made on a solution containing 1 × 10^−7^ M Pb(II), 6.3 × 10^−5^ M Cu(II), and 0.4 M HCl, with an accumulation potential of −0.7 V and a time of 60 s. As regards the pulse time, a range of 2–20 ms was investigated and it was found that the peak current decreased with increasing time. For further measurements, a pulse time of 2 ms was chosen as the most optimal one.

The effect of pulse amplitude was also investigated. The peak current was shown to increase with increasing amplitude up to about 160 mV, where there was a reversal of the relationship, and with further increases in the amplitude to 200 mV the signal decreased. The peak currents in the 120–160 mV amplitude range were comparable, and a pulse amplitude of 120 mV was selected for further study.

The effect of the scan rate was investigated in the range of 0.2 to 0.04 V/s, and because a change in the scan rate was observed to have no effect on the Pb(II) signal, a scan rate of 0.04 V/s was selected for further study.

### 3.6. Analytical Characteristics

**Figure 8:**
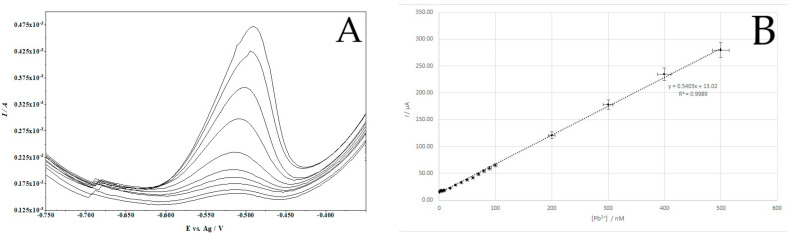
Example voltammograms (**A**) and the calibration curve (**B**). Scan rate 40 mV/s. Pulse time 2 ms and pulse height 120 mV.

Using the previously optimized parameters such as supporting electrolyte concentration, Cu(II) concentration, accumulation potential and time, as well as pulse time and height, measurements were carried out to obtain a calibration curve. A solution containing 6.3 × 10^−5^ M Cu(II) and 0.4 M HCl was prepared and increasing additions of Pb(II) were added to it. A calibration curve was made for an accumulation time of 120 s. A linear (correlation coefficient r = 0. 999) increase in the dependence of the lead peak current on the concentration of Pb(II) in the solution was found for the concentration range from 5 × 10^−10^ M to 5 × 10^−7^ M. Example voltammograms are presented in Figure 8A and the calibration curve is shown in Figure 8B. The equation of the calibration curve has the form y = 5 × 10^8^x + 13.02, where y is the peak current (µA) and x is the concentration of Pb(II) (M). For the developed procedure, the LOD was found to be 1.2 × 10^−10^ M, estimated from three times the standard deviation for the lowest Pb(II) concentration tested, while the LOQ was 3.6 × 10^−10^ M.

The precision of the electrode was assessed by repeatability tests (intra- and inter-day precision). The measurements were carried out for a solution with a concentration of 1 × 10^−8^ M. The RSD for six measurements taken back to back on the same electrode was 3.5%, while for six measurements made on six different electrodes taken day after day, it was 4.8%.

### 3.7. Interferences

**Figure 9:**
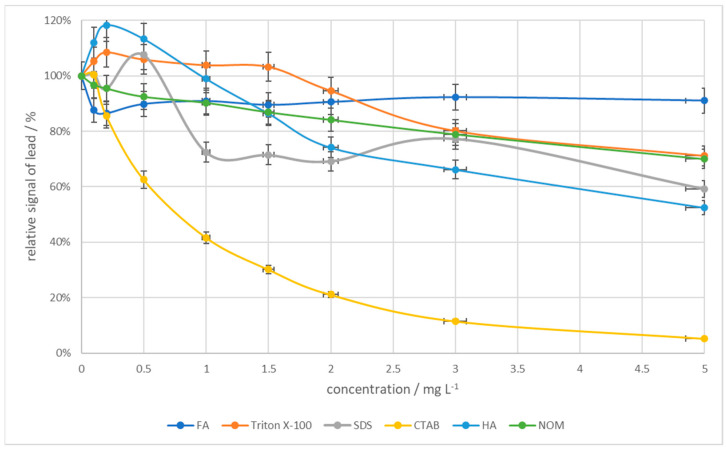
The influence of the interfering substances on the relative signal of 3 × 10^−8^ M Pb(II). Scan rate 40 mV/s. Pulse time 2 ms and pulse height 120 mV.

The impact of interfering substances such as Trition X-100, CTAB, SDS, FA, HA, and NOM was also studied. These substances represent different groups of chemical compounds found in surface waters: non-ionic surfactant, cationic surfactant, anionic surfactant, fulvic acids, humic acids, and natural organic matter, respectively. Solutions containing 3 × 10^−8^ M Pb(II), 6.3 × 10^−5^ M Cu(II), and 0.4 M HCl were prepared and the effects of the above-mentioned substances were checked one by one by adding more additives, checking the concentration range of 0.1–5 mg/L for each substance. As can be seen from Figure 9, fulvic acids had the smallest effect on the signal when added at a low concentration to the solution, causing the lead signal to decrease, but subsequent amounts added had virtually no effect on the signal. Triton X-100, SDS, HA, and NOM resulted in a peak reduction of about 40% at a concentration of 5 ppm. The presence of CTAB in the sample had the biggest impact on the proposed method, since it caused a sharp decrease in the signal until it almost disappeared. The relative lead signal in the presence of different concentrations of Trition X-100, CTAB, SDS, FA, HA, and NOM is shown in Figure 9.

The effect of other metal ions on the voltammetric Pb(II) signal was investigated using a solution containing fixed concentrations of Pb(II) and excesses of other metal ions. The result showed that an up to 100-fold excess of Al(III), Ca(II), Cr(III), Fe(III), Na(I), Mg(II), Ni(II), Hg(II), and Zn(II) did not have any significant effect on the Pb(II) peak current. The addition of a 100-fold excess of Cd(II) caused an approximately 60% decrease in the lead signal.

### 3.8. Analytical Application

To validate the developed procedure, tests were conducted using certified reference materials SPS-WW1 (waste water) and TM-25.5 (an environmental matrix). These materials were chosen for their composition, encompassing 13 different trace elements and Pb(II) at a concentration of 100 ng/mL in the case of SPS-WW1, and 27 other trace elements and Pb(II) at a concentration of 27 ng/mL in the case of TM-25.5. The remaining components in the solutions varied from 20 ng/mL to 2000 ng/mL, effectively reflecting the composition of environmental samples. All measurements were conducted using the standard addition method. The recoveries ranged from 90.15% to 100.95%, with the relative standard deviations ranging from 1.96% to 7.49%. The data from the recovery study using the certified materials are presented in Table 3. These figures confirm the good accuracy of the presented method.

Tests were also conducted on environmental samples to confirm the applicability of the method to such determinations. The voltammograms obtained for the water samples from the Bystrzyca River and Lake Piaseczno allow us to conclude that the lead content in these samples is below the detection limit. Therefore, the standard addition method was used and the recovery values were checked. Recovery values of 94.79% and 87.79% were obtained, respectively. Table 4 shows the data from the recovery study using the real samples.

## 4. Conclusions

As demonstrated in this paper, a copper film can be generated in situ on an MWCNTs/SPCE electrode. The study successfully developed and optimized a procedure for lead determination using a copper film-modified, carboxyl-functionalized, and multi-walled carbon nanotube-modified screen-printed carbon electrode (CuF/MWCNTs/SPCE) through anodic stripping voltammetry. The key parameters, such as electrolyte concentration, copper concentration, accumulation potential and time, as well as pulse amplitude and pulse time, were investigated and optimized. The optimal conditions were determined to be 0.4 M HCl and 6.3 × 10^−5^ M Cu(II) for the electrolyte concentration, with an accumulation potential of −0.7 V and an accumulation time of 120 s. Voltammetric stripping measurements were performed at a pulse amplitude of 120 mV and a pulse time of 2 ms. This is the first work on lead determination on a copper film-modified, carboxyl-functionalized, and multi-walled carbon nanotube-modified screen-printed carbon electrode (CuF/MWCNTs/SPCE). Compared with other voltammetric techniques, a very low detection limit for lead was obtained. Additionally, this method is simple and cheap, and does not require complicated apparatus. It can also be much quicker and cheaper than securing samples and transporting them to a laboratory since this method seems to make it possible to perform the analysis under field conditions.

## Figures and Tables

**Table 1:** Comparison of the procedures using ASV for the determination of Pb(II). The works are ranked according to the decreasing limit of detection.

Electrode	Linear Range [nM]	LOD [nM]	Influence of the Tested Organic Matrix	Real Sample	References
GCE	-	-	-	water	[31]
BDND	-	-	-	-	[32]
Bi/NA/SPGE	96.62–1449.28	14.49	-	water	[33]
CREs	13.53–531.40	13.53	-	tap water	[34]
3DrGO/F-BiNSs/ANE	40.00–600.00	12.50	-	coastal water	[35]
Nafion/MnCo_2_O_4_/GCE	48.31–3864.73	8.07	-	wine	[8]
HgF/GCE	48.31–222.22	4.30	-	tobacco	[13]
BiOCl/MWCNT/GCE	24.15–241.55	2.75	-	pore water	[36]
AgHgNpNf/GCE	48.31–579.71	1.16	-	human blood	[37]
Bi-PPy/MWCNT/CPE	0.53–724.64	0.48	-	tap water	[14]
GO-PEC/GCE	1.00–1000.00	0.40	-	biodiesel	[38]
Bi/Au-Gr-Cys/GCE	2.42–193.24	0.24	-	water	[39]
Cu/MWCNTs/SPCE	0.50–500.00	0.12	Triton X-100, SDS, CTAB, HA, FA, NOM	water	[this work]

GCE—glassy carbon electrode; BDND—boron-doped nanocrystalline diamond electrode; Bi/NA/SPGE—bismuth–nafion film screen-printed gold electrode; CRE—carbon rod electrode; 3DrGO/F-BiNSs—functional micro-needle electrode based on 3D reduced graphene oxide/flower-like bismuth nanosheets; Nafion/MnCo2O4/GCE—nafion–MnCo2O4 nanoparticle-modified glassy carbon electrode; HgF/GCE—mercury film-modified glassy carbon electrode; BiOCl/MWCNT/GCE—bismuth oxychloride particle–multiwalled carbon nanotube composite-modified glassy carbon electrode; AgHgNpNf/GCE—Ag–Hg nanoparticle–nafion-modified glassy carbon electrode; Bi-PPy/MWCNT/CPE—bismuth particle–polypyrrole film (PPy)-functionalized multiwalled carbon nanotube-modified carbon paste electrode; GO-PEC/GCE—graphene oxide–pectin-modified glassy carbon electrode; Bi/Au-Gr-Cys/GCE—gold nanoparticle–graphene–cysteine composite bismuth film-modified glassy carbon electrode.

**Table 2:** The EDS analysis results obtained for the unmodified CuF/MWCNTs/SPCE electrode and after modification with a copper film.

Element	Percentage of Individual Elements for the Electrode before and after Modification with a Copper Film			
	Electrode before Modification		Electrode after Modification	
	Weight [%]	Atomic [%]	Weight [%]	Atomic [%]
C	85.18	90.91	87.20	93.14
O	8.47	6.79	5.21	4.18
S	0.15	0.06	0.15	0.06
Cl	6.19	2.27	6.97	2.52
Cu	-	-	0.47	0.09

**Table 3:** The analytical results of Pb(II) determination in the certified reference materials SPS-WW1 and TM-25.5. The samples were examined using the standard addition method.

Certified Reference Material	Pb(II) Content [µg/L]	Pb(II) Found [µg/L]	Recovery [%]	RSD (n = 3) [%]	*t*-Test
SPS-WW1	100.0 ± 0.5	90.24	90.15	4.5	0.77
		93.51	93.42	4.2	0.05
		97.37	97.27	3.5	1.05
TM-25.5	27.0 ± 2.4	24.38	92.36	3.0	1.21
		26.65	100.95	5.3	0.93
		25.00	94.68	4.3	0.31

**Table 4:** The analytical results of Cd(II) determination in natural water samples. The samples were examined using the standard addition method.

Samples	Pb(II) Added [nmol/L]	Pb(II) Found [nmol/L]	Recovery [%]	RSD (n = 3) [%]
Bystrzyca River	10	9.13	91.25	4.9
		9.20	92.04	5.1
		8.78	87.79	4.1
Lake Piaseczno		9.48	94.79	3.3
		9.34	93.44	4.6
		9.14	91.36	3.1

## Data Availability

Data are contained within the article.
